# Psychosocial stress impairs health behavior in patients with mental disorders

**DOI:** 10.1186/s12888-018-1956-8

**Published:** 2018-12-04

**Authors:** Till Fabian Beutel, Rüdiger Zwerenz, Matthias Michal

**Affiliations:** 1 Department of Psychosomatic Medicine and Psychotherapy, University Medical Center of the Johannes Gutenberg University Mainz, Untere Zahlbacher Str. 8, 55131 Mainz, Germany; 2 Institute of Occupational, Social and Environmental Medicine, University Medical Center of the Johannes Gutenberg University Mainz, Obere Zahlbacher Str. 67, 55131 Mainz, Germany

## Abstract

**Background:**

It has been shown, that in the general population psychosocial stress affects health behaviors. However similar studies of high risk populations are sparse. Therefore, the aim of this cross-sectional study is to analyze the association between common psychosocial stressors and health behavior in a sample of patients with mental disorders.

**Methods:**

We analyzed data of n = 2326 outpatients from a mental health care department. Severity of psychosocial stress was assessed by the PHQ-stress module of the Patient Health Questionnaire (PHQ). Health behaviors included obesity, uncontrolled eating, smoking and physical inactivity. Multiple binary regression models were conducted for the PHQ-stress score and for each of the ten PHQ-stress items as independent variables.

**Results:**

'Financial stress' and 'having no one to turn to with problems' were mainly associated with adverse health behaviors after adjustment for multivariate effects. The most affected health behaviors were uncontrolled eating in both sexes and obesity in women.

**Conclusion:**

Our findings indicate specific influences of psychosocial stressors on unhealthy behaviors in a clinical sample. Patients with financial strain and lack of social support might need specific support for improving their health behavior.

## Background

Stress is widespread and about a quarter of the US citizens rate their stress levels as “extreme” (8, 9 or 10 on a 10-point scale), with money, work and family responsibilities being the top sources of stress [1]. The concept of stress and its assessment have been viewed from a biomedical, psychological, sociological and environmental perspective [2–4]. Thus it can be operationalized differently and psychosocial stress has been considered for instance as major life events, chronic strains, day-to-day hassles and also as trauma [5].

Commonly analyzed outcomes of stress concern physical and mental health [6], such as cardiovascular disease [7, 8], cancer [9] or depression and anxiety [10, 11].

The complex pathways linking stress to disease and dysfunction are not fully understood [6]. Psychological stress has been linked to health behavior [12–14], which has been demonstrated to be a potential link to health outcomes [15]. Many studies have focused on the relationship between work-related stress and unhealthy lifestyles such as smoking or physical inactivity in healthy subjects [16–19].

The aim of our study is to explore the association between stress factors and health behavior in a clinical sample. Previous research has found significant correlations between stress and health behavior which will be reported below for selective forms. Most studies analyzed subjects from the general population.

### Psychosocial stress and health behavior

#### Smoking

A longitudinal study investigated how psychosocial stressors (e. g. relationship stress, work stress, financial stress and family problems) influence smoking behavior in a general US sample over 9-10 years [12]. After adjustment for age, sex and socioeconomic status, Slopen et al. (2013) found an association between high psychosocial stress and persistence of smoking. However, there is a need for more research concerning the impact of certain psychosocial stressors on smoking behavior [12]. Regarding sex issues, it has been found that women were more vulnerable to tobacco use when having stress [20]. Female smokers had greater craving, arousal and stress reactions when confronted with stress cues, compared to male smokers [21, 22].

Different mechanisms are possible by which smoking behavior is influenced through stress. Smoking desire can be activated by acute psychosocial stress [13, 21], which has been shown for the Trier Social Stress Test (TSST) [13] and it can be used as a dysfunctional way to cope with stress [12, 23, 24]. Alternatively, self-regulation can be diminished as a result of stress which leads to smoking [25, 26].

#### Obesity and eating behavior

The amount of stress in life was associated with obesity in a large sample (N > 112.000) of the general Canadian population in a cross-sectional study [27]. A further study indicated that more than three chronic stressors compared to no stressor were linked to significantly higher odds of obesity (OR = 1.5) and percentage body fat (ß = 1.5) in a Hispanic sample [28]. Harding et al. (2014) [29] analyzed subjects from the general Australian population over five years. Psychosocial stressors were significantly associated with weight gain, but not with weight loss [29]. An association between psychosocial stress and weight gain has also been found in a longitudinal study in the general US population [14]. Relevant stressors were financial problems (difficulty paying bills) and job-related demands in both sexes, in women additionally constraints in life and family strains. Chen & Qian (2012) found a stronger association between stress and obesity in women in their Canadian sample [27].

Psychosocial stress is also associated with more uncontrolled eating [1, 30] and food craving which influences body weight [31]. Only few studies have considered which certain stressors influence eating patterns. Work-related or interpersonal (e.g. family problems, argument with partner) hassles have been associated with unhealthy eating behavior [32]. Significant predictors in terms of binge eating were as different stressors as changes in family or relationship, experiences of abuse, work stress or critical comments about weight or shape [33] and bereavement or separation from a family member [34].

More research is necessary to identify groups that are vulnerable to obesity and dysfunctional eating patterns when being under stress [28].

#### Physical inactivity

Although measures of stress were different from each other and the quality of the studies varied, in a review [35] a mainly negative impact of stress on physical activity has been found. The authors also reported of some studies that had found a positive impact on physical activity, as some individuals are physical active to cope with stress. A systematic review [36] reported of typical barriers for physical activity. Those were stress and low mood in patients with mental illness. Motivating aspects for physical activity on the other side were reducing stress and improving mood.

#### Aims and hypothesis

In our study, we want to analyze the association between psychosocial stress and health behavior in patients seeking psychotherapeutic treatment. Moreover, we want to figure out which specific psychosocial stressors are relevant for certain dysfunctional health behaviors. Previous research has found sex differences concerning the impact of stress on health behavior in the general population for instance in terms of smoking [20–22] and obesity [27], whereas women seemed to be more vulnerable. For that reason we want to take sex differences into account. Based on previous literature we suppose to see higher correlations between psychosocial stress and health behaviors in women. To the best of our knowledge this is the first study to analyze the association between psychosocial stress, assessed by the stress module of PHQ, and health behavior in a clinical sample.

## Method

### Procedure and study sample

We analyzed the medical records of outpatients who had been examined and treated in the Department of Psychosomatic Medicine and Psychotherapy at a university medical center between 01/2010 and 12/2013. Data of N = 2326 patients who were diagnosed and who completed the measures went into the analyses. Informed consent was obtained. The work has been carried out in accordance with the Declaration of Helsinki. The publication of this work has been approved by the Ethics Committee of the State Board of Physicians of Rhineland-Palatinate, Mainz, Germany.

### Measures

The medical records contain a comprehensive set of measures which are filled out routinely by patients and by the attending physician or clinical psychologist. The self-reported measures include socio-demographic items, health behaviors and the Patient Health Questionnaire (PHQ-D, German Version). All patients were diagnosed by a clinician according to ICD-10 Chapter V diagnoses.

### Health Behaviors

Physical inactivity was determined by one item, which had previously predicted mortality in patients with a history of infarction or cardiovascular disease [37]. The degree of physical activity during the last month (“activities such as 15 to 20 minutes of brisk walking, swimming, general conditioning or recreational sports”) was rated according to a 5-point Likert scale: “not at all active” or “a little active (1-2 times per month)” (0), “fairly active (3-4 times per month)” (1), “quite active (1-2 times per week)” (2), “very active (3-4 times per week)” (3) and “extremely active (more than 5 times a week)” (4) [37]. Patients who rated physical activity of 15-20 minutes 3-4 times per month or less were classified as physical inactive. Smoking was asked dichotomously (yes / no). The amount of cigarettes per day was recorded in smokers (continuous). Obesity was categorized according to the body mass index (BMI ≥ 30.0). Control about the amount of food ingested was measured by an item of PHQ-D section assessing symptoms of “binge eating”.

#### Patient Health Questionnaire - stress scale

Stress factors were assessed using the section “PHQ-stress” from the PHQ-D (German Version, [38]). It measures psychosocial strain during the last month by ten items including health, work/financial, social and traumatic stress. Ratings comprise “not at all bothered” (0), “bothered a little” (1) and “bothered a lot” (2). The summation shows cumulative values between “0” and “20” which represent the severity of stress. While validated cut-off scores are available for other scales of PHQ-D [39], no cut-off scores exist for PHQ-stress. We calculated Cronbach’s alpha for the PHQ-stress scale, which was acceptable (α = 0.71).

### Statistical Analyses

Frequencies and descriptive statistics were calculated for all variables. Reliability analysis (Cronbach’s alpha) was conducted for the PHQ-stress scale. The relationship between psychosocial stressors and health behavior was calculated using Chi^2^-test (nominal / ordinal variables) and F-Test for continuous variables. Effect sizes were calculated using Cohen’s d for means and Cramer’s V for crosstabs. We computed binary logistic and linear regression analyses (method enter) with health behaviors as dependent variables. Variables were eligible to be entered into the regression model when being significantly associated with health behavior in former analyses. Regression analyses were conducted unadjusted and adjusted to check for an incremental effect of stress factors on health behaviors beyond known variables. Where necessary we adjusted multiple regression analyses for depressive disorders. We categorized depression as “0” (no depressive disorder), “1” (mild depressive disorder; ICD-10 F32.0, F33.0, F31.3), “2” (moderate depressive disorder; ICD-10 F32.1, F33.1) and “3” (severe depressive disorder; ICD-10 F32.2, F33.2). Analyses were additionally adjusted for strong overweight of parents, mainly concerning obesity and uncontrolled eating.

We conducted analyses for the PHQ-stress score and for each of the ten PHQ-stress items. All analyses were stratified by sex. Statistical analyses were carried out using IBM SPSS Statistics 23. Significance level was defined as p < 0.05.

## Results

### Demographic variables & health behavior

Table 1 shows descriptive information. The sample consisted of 56.6% (*n* = 1316) women, the mean age was 39.5 years (SD = 14.1, range = 18-93 years).

**Table 1:** Demographic and clinical characteristics, health behavior

	Total	Women	Men	*p*-value
Age Mean (SD; range)	39.5 (14.1; 18-93)	40.4 (14.2; 18-93)	38.3 (13.9; 18-83)	**<0.001**
Sex (%; *n*)		56.6 (1316)	43.4 (1010)	
Marital status (%; *n*)				**<0.001**
single	50,3 (1133)	46.3 (589)	55.6 (544)	
married	33,1 (745)	33.9 (431)	32,1 (314)	
separate living	3,8 (86)	4.4 (56)	3.1 (30)	
divorced	10,4 (234)	11.9 (152)	8.4 (82)	
widowed	2,2 (49)	3.1 (40)	0.9(9)	
School degree (%; *n*)				n.s.
in school	1.2 (27)	0.9 (11)	1.6 (16)	
no or special school degree	1.9 (43)	1.8 (23)	2.0 (20)	
basic school (“Hauptschule”)	24.3 (548)	22.8 (292)	26.2 (256)	
secondary school (“Realschule”)	29.0 (654)	32.4 (415)	24.4 (239)	
grammar school (“Abitur”)	41.7 (941)	40.3 (515)	43.6 (426)	
Vocational degree (%; *n*)				**<0.001**
no vocational degree	15.3 (329)	12.3 (149)	19.2 (180)	
still in vocational training	10.2 (218)	10.1 (122)	10.2 (96)	
apprenticeship	39.3 (844)	43.6 (526)	33.9 (318)	
master craftsman	3.4 (72)	1.7 (20)	5.5 (52)	
university degree	21.0 (451)	20.5 (248)	21.6 (203)	
others	10.8 (232)	11.8 (142)	9.6 (90)	
Vocational position (%; *n*)				**<0.001**
employed full time	33.4 (743)	26.4 (333)	42.4 (410)	
employed part time	16.3 (363)	23.4 (295)	7.0 (68)	
not employed (e.g. housewife)	5.8 (130)	9.5 (120)	1.0 (10)	
vocational training	8.6 (191)	7.9 (100)	9.4 (91)	
unemployed	14.1 (315)	10.8 (136)	18.5 (179)	
retired	9.7 (217)	10.6 (133)	8.7 (84)	
others	12.0 (268)	11.3 (143)	12.9 (125)	
Diagnoses ICD-10 (%; *n*)				
depression (F32, F33, F34) (%)				**<0.001**
no depressive disorder / other	50.0 (1162)	46.4 (610)	54.7 (552)	
mild depressive disorder	8.0 (185)	8.5 (112)	7.2 (73)	
moderate depressive disorder	34.6 (804)	37.1 (488)	31.3 (316)	
severe depressive disorder	7.5 (175)	8.1 (106)	6.8 (69)	
anxiety disorder (F40. F41) (%)	27.3 (634)	29.0 (382)	25.0 (252)	**<0.05**
somatoform disorder (F45) (%)	18.2 (423)	20.7 (272)	15.0 (151)	**<0.001**
number of F-diagnoses/patient	2.0 (2326)	2.0 (1316)	2.0 (1010)	
Health behavior				
smoking (% yes; *n*)	38.4 (851)	34.0% (424)	44.0% (427)	**<0.001**
number of cig./day (M; SD)	11.4 (15.7)	10.5 (16.5)	12.4 (14.9)	**<0.05**
BMI (M; SD)	25.5 (5.8)	24.9 (6.3)	26.2 (5.1)	**<0.001**
obesity (BMI ≥ 30.0) (%; *n*)	18.5 (377)	19.3 (218)	17.4 (159)	
uncontrolled eating (% yes; *n*)	24.9 (552)	27.3 (341)	21.7 (211)	**<0.001**
physical inactivity (< 2)	61.9 (1345)	62.3 (762)	61.3 (583)	

Significant differences between female (*n* = 1316) and male (*n* = 1010) patients were tested by Chi^2^-Test for crosstabs and by F-Test for means, significant differences are highlighted in bold and presented as p-value

38.4% (*n* = 851) of the patients were smokers, among those were significantly (*p* < 0.001) more male (44.0%) compared to female smokers (34.0%). Male smokers consumed a higher number of cigarettes per day (*p* < 0.05). The body mass index (BMI) was higher in men (*p* < 0.001), but women were slightly more likely to be obese (BMI ≥ 30; n. s.). The prevalence of physical inactivity was around 62% (see Table 1).

### PHQ-stress

The mean PHQ-stress score was M = 8.08 (SD = 4.26), women had significantly (p < 0.001) higher scores than men (see Table 2). The most frequent stressor was *worries about health* affecting about 57% of the patients a lot (women: 61.8%; men: 51.0%; p < 0.001). About one third of the patients were bothered a lot by *stress at work or at school* (34.3%), *worries about weight or look* (32.1%) and *financial problems or worries* (30.2%). *Worries about weight or look* were the second most prevalent stressors in women, *financial problems or worries* in men. Male patients were slightly more often bothered a lot by *financial problems* (n. s.), although women were significantly more bothered by all other psychosocial stressors, in terms of *difficulties with partner* only by trend (n. s.). The third most severe stressor was *stress at work or at school*, concerning both men and women.

**Table 2:** Frequency of psychosocial stress stratified by sex

PHQ-stress scale (M; SD)	M	SD						*p*-value
Total	8.08	4.26						**<0.001**
Women	8.70	4.29						
Men	7.34	4.11						
PHQ-stress items (M; *n*)								
	not bothered		bothered a little		bothered a lot			
Worries about health	15.1	(335)	27.8	(619)	57.1	(1269)		**<0.001**
Women	12.2	(152)	26.0	(325)	61.8	(772)	[1]	
Men	18.8	(183)	30.2	(294)	51.0	(497)	[1]	
Stress at work or at school	43.7	(933)	22.0	(470)	34.3	(732)		**<0.01**
Women	41.9	(501)	20.5	(245)	37.6	(449)	[3]	
Men	46.0	(432)	23.9	(225)	30.1	(283)	[3]	
Worries about weight or look	31.9	(710)	36.0	(802)	32.1	(716)		**<0.001**
Women	27.3	(343)	33.0	(415)	39.6	(498)	[2]	
Men	37.8	(367)	39.8	(387)	22.4	(218)	[6]	
Financial problems or worries	39.6	(877)	30.1	(667)	30.2	(669)		n.s.
Women	41.7	(519)	29.0	(361)	29.4	(366)	[5]	
Men	37.0	(358)	31.6	(306)	31.3	(303)	[2]	
Little or no sexual desire	46.4	(1004)	26.7	(577)	26.9	(582)		**<0.01**
Women	45.1	(543)	24.8	(299)	30.1	(363)	[4]	
Men	48.1	(461)	29.0	(278)	22.9	(219)	[5]	
Difficulties with partner	49.7	(1066)	25.5	(546)	24.8	(532)		n.s.
Women	48.7	(584)	25.1	(301)	26.1	(313)	[7]	
Men	51.0	(482)	25.9	(245)	23.2	(219)	[4]	
Thinking / dreaming about something terrible that happened	56.2	(1236)	18.9	(416)	24.8	(546)		**<0.001**
Women	49.1	(606)	21.6	(266)	29.3	(361)	[6]	
Men	65.3	(630)	15.5	(150)	19.2	(185)	[8]	
Having no one to turn to with problems	47.2	(1041)	29.8	(657)	23.0	(507)		**<0.05**
Women	45.0	(558)	30.4	(377)	24.7	(306)	[8]	
Men	50.1	(483)	29.0	(280)	20.9	(201)	[7]	
Something bad that happened recently	64.4	(1391)	14.7	(317)	20.9	(452)		**<0.001**
Women	60.8	(736)	15.2	(184)	24.0	(291)	[9]	
Men	69.0	(655)	14.0	(133)	17.0	(161)	[9]	
Stress of care taking	61.5	(1336)	20.5	(446)	18.0	(392)		**<0.001**
Women	57.3	(700)	21.6	(264)	21.0	(257)	[10]	
Men	66.7	(636)	19.1	(182)	14.2	(135)	[10]	

Psychosocial stressors are rated “not at all bothered” to “bothered a lot” and presented according to frequency of “bothered a lot”, rankings of men and women are presented in square brackets. Sex differences were calculated by Chi^2^-Test. Significant differences are highlighted in bold

### Associations between PHQ-stress and health behaviors

#### BMI and obesity

The mean stress score was weakly associated (r = 0.09; *p* < 0.001) with BMI (continuous) (see Table 3). Obese (BMI ≥ 30) patients had a higher stress score (*p* < 0.001). Stratified for sex, higher overall stress scores were associated with obesity only in women (see Table 4). This was also significant after adjustment for confounders (adj. OR = 1.07; *p* < 0.01). On item level *worries about weight or look* (adj. OR = 2.02; *p* < 0.001), *financial problems or worries* (adj. OR = 1.64; *p* < 0.01) and *having no one to turn to with problems* (adj. OR = 1.55; *p* < 0.05) were significant predictors for obesity in women. In men *worries about weight or look* (adj. OR = 3.18; *p* < 0.001) predicted obesity in an adjusted logistic regression model (see Table 4).

**Table 3:** Association between PHQ-stress and health behavior

	BMI					Obesity (BMI ≥ 30)			Uncontrolled eating			Smoking			Cig / day					Physical inactivity		
						no	yes		no	yes		no	yes							no	yes	
PHQ-stress scale (0-20)	r=0.09***					M=7.92	M=8.94	***	M=7.43	M=10.16	***	M=7.85	M=8.47	**	r=0.08**					M=7.40	M=8.52	***
						SD=4.22	SD=4.33		SD=4.13	SD=3.98		SD=4.13	SD=4.37							SD=4.06	SD=4.28	
	BMI (M, SD)					Obesity (% BMI ≥ 30)			Uncontrolled eating (% yes)			Smoking (% yes)			Cig / day (M, SD)					Physical inactivity (% inactive)		
PHQ-stress items (dichotomous)	not/a little bothered		bothered a lot			not/a little bothered	bothered a lot		not/a little bothered	bothered a lot		not/a little bothered	bothered a lot		not/a little bothered		bothered a lot			not/a little bothered	bothered a lot	
					d_Cohen_			Cramérs V			Cramérs V			Cramérs V					d_Cohen_			Cramérs V
Worries about health	25.3	(5.4)	25.6	(6.1)		**16.2**	**19.9**	0.05*	**22.3**	**26.8**	0.05*	**43.4**	**34.9**	0.09***	11.2	(12.6)	10.7	(15.5)		**58.5**	**64.5**	0.06**
Stress at work or at school	25.3	(5.8)	25.6	(5.9)		**16.6**	**20.6**	0.05*	**20.8**	**31.9**	0.12***	40.1	35.8		**11.5**	**(15.3)**	**9.7**	**(12.9)**	0.12*	61.7	62.4	
Worries about weight or look	**25.1**	**(4.9)**	**26.4**	**(7.3)**	0.23***	**14.3**	**28.0**	0.16***	**15.2**	**45.6**	0.33***	38.7	38.4		10.8	(13.3)	11.5	(16.5)		60.7	64.5	
Financial problems or worries	**25.1**	**(5.8)**	**26.3**	**(6.0)**	0.21***	**16.0**	**24.3**	0.10***	**20.7**	**34.4**	0.15***	**32.8**	**51.3**	0.18***	**9.8**	**(14.7)**	**13.3**	**(13.7)**	0.24***	**59.0**	**68.0**	0.09***
Little or no sexual desire	25.4	(5.7)	25.6	(6.1)		18.6	17.9		**22.8**	**29.6**	0.07**	39.2	37.3		10.9	(14.7)	11.5	(14.1)		60.8	65.5	
Difficulties with partner	25.5	(5.8)	25.3	(5.8)		18.7	16.5		**22.4**	**30.4**	0.08***	**36.8**	**44.4**	0.07**	10.9	(15.4)	11.4	(11.7)		61.0	65.3	
Thinking / dreaming about sth terrible	**25.2**	**(5.5)**	**26.4**	**(6.7)**	0.21***	**17.0**	**22.7**	0.06**	**21.0**	**36.6**	0.16***	37.4	40.7		10.5	(13.1)	11.8	(16.0)		60.9	64.7	
Having no one to turn to with problems	25.3	(5.7)	25.9	(6.3)		**17.3**	**22.1**	0.05*	**21.9**	**34.8**	0.13***	37.7	40.6		10.7	(14.5)	11.7	(14.3)		**58.7**	**72.0**	0.12***
Something bad that happened recently	25.4	(6.0)	25.7	(5.2)		17.9	19.6		24.7	25.7		37.9	40.6		10.7	(13.8)	11.9	(16.7)		**60.4**	**66.9**	0.06*
Stress of care taking	**25.3**	**(5.7)**	**26.3**	**(6.6)**	0.17**	**17.3**	**23.2**	0.06**	**22.7**	**34.3**	0.10***	37.9	42.8		10.7	(14.7)	12.4	(13.5)		**60.8**	**68.0**	0.06**

***p < 0.001; **p < 0.01; *p < 0.05; correlation analyses *r* Pearson’s r; *M* mean, *SD* standard deviation. PHQ-stress items: comparison between “not or a little bothered” vs. “bothered a lot”. Physical inactivity: patients who rated 3-4 times per month of physical activity or less were classified as physical inactive. Significance level was tested by Chi^2^ for crosstabs and F-Test for means

Effect sizes for significant results were calculated using Cohen’s d for means and Cramer’s V for crosstabs. Significant results are highlighted in bold

**Table 4:** Multiple regression analyses (binary, linear) between PHQ-stress and health behavior stratified by sex

	Obesity			Uncontrolled eating			Smoking			Cig/day		Physical inactivity		
PHQ-stress - Women	OR	95% CI	Nagelk. R^2^	OR	95% CI	Nagelk. R^2^	OR	95% CI	Nagelk. R^2^	stand. ß	adj. R^2^	OR	95% CI	Nagelk. R^2^
PHQ-stress scale (0-20) (unadj.)	1.07**	**1.03-1.11**	**2.0**	1.18***	**1.14-1.23**	**13.1**	1.05**	**1.02-1.08**	**1.3**	n.s.		1.06***	**1.03-1.09**	**1.9**
PHQ-stress scale (0-20) (adjusted)	1.07**	**1.03-1.11**	**11.7**	1.17***	**1.12-1.22**	**18.5**	1.03	1.00-1.07	n.s.	n.s.		1.05*	**1.01-1.08**	**5.5**
PHQ-stress items (dichotomous)														
Worries about health (unadj.)	n.s.			n.s.			0.77*	**0.59-0.83**	**0.5**	n.s.		n.s.		
adjusted	n.s.			n.s.			0.77	0.57-1.03	n.s.	n.s.		n.s.		
Stress at work or at school (unadj.)	n.s.			1.75***	**1.35-2.27**	**2.2**	n.s.			n.s.		n.s.		
adjusted	n.s.			1.69***	**1.26-2.27**	**10.4**	n.s.			n.s.		n.s.		
Worries about weight or look (unadj.)	2.03***	**1.51-2.74**	**3.0**	4.86***	**3.72-6.36**	**15.9**	n.s.			n.s.		n.s.		
adjusted	2.02***	**1.46-2.79**	**12.6**	4.49***	**3.38-5.97**	**21.7**	n.s.			n.s.		n.s.		
Financial problems or worries (unadj.)	1.78***	**1.30-2.43**	**1.9**	2.15***	**1.65-2.79**	**3.7**	1.85***	**1.43-2.38**	**2.5**	0.10**	**0.9**	1.48***	**1.22-1.80**	**1.0**
adjusted	1.64**	**1.18-2.31**	**11.3**	1.79***	**1.34-2.38**	**11.0**	1.45*	**1.08-1.93**	**14.1**	0.09*	**1.9**	1.34*	**1.01-1.77**	**4.1**
Little or no sexual desire (unadj.)	n.s.			1.39*	**1.06-1.82**	**0.7**	n.s.			n.s.		n.s.		
adjusted	n.s.			1.30	0.97-1.74	n.s.	n.s.			n.s.		n.s.		
Difficulties with partner (unadj.)	n.s.			1.59**	**1.20-2.10**	**1.2**	1.44**	**1.10-1.89**	**0.8**	n.s.		1.40*	**1.06-1.85**	**0.7**
adjusted	n.s.			1.36	0.99-1.87	n.s.	1.47*	**1.08-1.99**	**14.3**	n.s.		1.30	0.97-1.76	n.s.
Thinking/dreaming about sth terrible that happened (unadj.)	1.46*	**1.06-2.00**	**0.8**	1.98***	**1.52-2.59**	**2.9**	1.56***	**1.20-2.01**	**1.3**	0.12**	**1.2**	n.s.		
adjusted	1.37	0.98-1.92	n.s.	1.78***	**1.34-2.37**	**10.9**	1.52**	**1.14-2.03**	**15.0**	0.10**	**2.7**	n.s.		
Having no one to turn to with problems (unadj.)	1.71**	**1.24-2.37**	**1.5**	1.96***	**1.49-2.59**	**2.6**	n.s.			n.s.		1.67***	**1.26-2.23**	**1.5**
adjusted	1.55*	**1.10-2.19**	**10.9**	1.58**	**1.17-2.15**	**10.1**	n.s.			n.s.		1.50**	**1.10-2.05**	**4.9**
Something bad that happened recently (unadj.)	n.s.			n.s.			1.39*	**1.06-1.83**	**0.6**	n.s.		1.33*	**1.06-1.66**	**0.9**
adjusted	n.s.						1.26	0.91-1.74	n.s.	n.s.		1.40*	**1.04-1.89**	**4.5**
Stress of care taking (unadj.)	1.46*	**1.03-2.07**	**0.6**	1.58**	**1.17-2.13**	**1.0**	n.s.			n.s.		n.s.		
adjusted	1.41	0.97-2.06	n.s.	1.57**	**1.13-2.18**	**9.9**	n.s.			n.s.		n.s.		
PHQ-stress - Men	OR	95% CI	Nagelk. R^2^	OR	95% CI	Nagelk. R^2^	OR	95% CI	Nagelk. R^2^	stand. ß	adj. R^2^	OR	95% CI	Nagelk. R^2^
PHQ-stress scale (0-20) (unadj.)	1.03	0.99-1.08	n.s.	1.13***	**1.09-1.18**	**6.7**	1.04*	**1.00-1.07**	**0.7**	0.12**	**1.2**	1.07***	**1.04-1.11**	**2.6**
PHQ-stress scale (0-20) (adj.)	1.02	0.98-1.07	n.s.	1.14***	**1.09-1.19**	**9.3**	1.06**	**1.02-1.10**	**19.8**	0.10*	**5.7**	1.06**	**1.02-1.10**	**5.2**
PHQ-stress items (dichotomous)														
Worries about health (unadj.)	n.s.			n.s.			0.69**	**0.53-0.89**	**1.2**			1.45**	**1.11-1.89**	**1.1**
adjusted	n.s.			n.s.			0.73*	**0.55-0.98**	**19.3**			1.33*	**1.01-1.75**	**4.4**
Stress at work or at school (unadj.)	n.s.			1.72**	**1.24-2.39**	**1.7**	n.s.			n.s.		n.s.		
adjusted	n.s.			1.57**	**1.11-2.21**	**3.9**	n.s.			n.s.		n.s.		
Worries about weight or look (unadj.)	2.89***	**1.99-4.18**	**5.4**	4.27***	**3.05-5.97**	**10.9**	n.s.					n.s.		
adjusted	3.18***	**2.15-4.71**	**13.8**	4.05***	**2.88-5.68**	**12.8**	n.s.					n.s.		
Financial problems or worries (unadj.)	1.57*	**1.10-2.24**	**1.1**	1.87***	**1.36-2.57**	**2.3**	2.59***	**1.96-3.43**	**6.2**	0.13***	**1.5**	1.49**	**1.11-1.99**	**1.1**
adjusted	1.39	0.95-2.04	n.s.	1.75***	**1.26-2.41**	**4.5**	2.25***	**1.63-3.09**	**22.4**	0.12**	**6.5**	1.30	0.96-1.76	n.s.
Little or no sexual desire (unadj.)	n.s.			**1.41**	**0.99-2.01**	**0.6**	n.s.			n.s.		n.s.		
adjusted	n.s.			1.37	0.94-1.99	n.s.	n.s.			n.s.		n.s.		
Difficulties with partner (unadj.)	n.s.			n.s.			n.s.			n.s.		n.s.		
adjusted	n.s.			n.s.			n.s.			n.s.		n.s.		
Thinking/dreaming about sth terrible that happened (unadj.)	n.s.			2.33***	**1.63-3.33**	**3.3**	n.s.			n.s.		n.s.		
adjusted	n.s.			2.19***	**1.51-3.17**	**5.5**	n.s.			n.s.		n.s.		
Having no one to turn to with problems (unadj.)	n.s.			1.76**	**1.23-2.51**	**1.5**	n.s.			n.s.		2.02***	**1.42-2.88**	**2.4**
adjusted	n.s.			1.6*	**1.11-2.31**	**3.6**	n.s.			n.s.		1.87***	**1.30-2.69**	**5.2**
Something bad that happened recently (unadj.)	n.s.			n.s.			n.s.			n.s.		n.s.		
adjusted	n.s.			n.s.			n.s.			n.s.		n.s.		
Stress of care taking (unadj.)	n.s.			2.03***	**1.36-3.03**	**1.9**	1.59*	**1.10-2.31**	**0.9**	0.09*	**0.6**	1.72**	**1.14-2.60**	**1.0**
adjusted	n.s.			2.00***	**1.33-3.03**	**4.3**	1.87**	**1.22-2.87**	20.2**	0.11*	**6.1**	1.64*	**1.08-2.51**	**4.2**

Each PHQ-stress item was dichotomized in “not or a little bothered (0)” and “bothered a lot (1)”. PHQ-stress scale ranges from 0-20. Logistic Regression analyses are presented unadjusted and adjusted for age, education, vocational training, depression

Analyses concerning obesity (BMI>30) and binge eating were adjusted for obesity of parents. Non-significant results from previous analyses were not considered in regression analyses and marked as n.s. *unadj* unadjusted. ***p < 0.001; **p < 0.01; *p < 0.05. *OR* Odds Ratio. Effect sizes (Cohen’s d) are calculated for OR. Nagelk. R^2^ = explained variance (in %). Significant results are highlighted in bold

#### Uncontrolled eating

Higher overall stress scores were associated with a higher chance of uncontrolled eating in women (adj. OR = 1.17; *p* < 0.001) and in men (adj. OR = 1.14; *p* < 0.001) in an adjusted logistic regression model (see Table 4). Women and men showed higher odds of uncontrolled eating when being bothered a lot by *worries about weight or look* (women: adj. OR = 4.49; p < 0.001; men: adj. OR = 4.05; *p* < 0.001), *financial problems* (women: adj. OR = 1.79; *p* < 0.001; men: adj. OR = 1.75; *p* < 0.001), *having no one to turn to with problems* (women: adj. OR = 1.58; *p* < 0.01; men: adj. OR = 1.60; *p* < 0.01), *stress of care taking* (women: adj. OR = 1.57; *p* < 0.01; men: adj. OR = 2.00; *p* < 0.001), *stress at work or at school* (women: adj. OR = 1.69; *p* < 0.001; men: adj. OR = 1.57; *p* < 0.01) and *thinking/dreaming about something terrible that happened* (women: adj. OR = 1.78; *p* < 0.001; men: adj. OR = 2.19; *p* < 0.001).

#### Smoking and cigarettes per day

Patients with higher stress scores were more likely to be smokers (*p* < 0.01; see Table 3). The overall stress score was - adjusted for multivariate effects - only significant in male patients (see Table 4). Being bothered a lot by *worries about health* had lower odds towards smoking in male (adj. OR = 0.73; *p* < 0.05) and in female patients (adj. OR = 0.77; n. s.). Patients with severe *financial problems* were more likely to smoke, the odds ratio was higher in men (adj. OR = 2.25; *p* < 0.001) compared to women (adj. OR = 1.45; *p* < 0.05). Moreover, male and female smokers fumed significantly more cigarettes when being bothered a lot by *financial problems* (women: stand. ß = 0.09; *p* < 0.05; men: stand. ß = 0.12; *p* < 0.01). *Difficulties with partner* (adj. OR = 1.47; *p* < 0.05) and *thinking/dreaming about something terrible that happened* (adj. OR = 1.52; *p* < 0.01) were further predictors in women. Men were significantly more likely to be smoking when being bothered a lot by *stress of care taking* (adj. OR = 1.87; *p* < 0.01), which was also associated with a higher consumption of cigarettes (stand. ß = 0.11; *p* < 0.05).

#### Physical inactivity

Higher overall stress scores went along with a higher prevalence of physical inactivity, which was significant as a mean score (*p* < 0.001, see Table 3) and in an adjusted regression model for men (*p* < 0.01) and women (*p* < 0.05; see Table 4). On item-level, both male and female patients had a higher prevalence of physical inactivity when being bothered a lot by *having no one to turn to with problems* (men: adj. OR = 1.87; *p* < 0.001; women: adj. OR = 1.50; *p* < 0.01). Men also showed a higher prevalence of physical inactivity when being bothered a lot by *stress of care taking* (adj. OR = 1.64; *p* < 0.05) and *worries about health* (adj. OR = 1.33; *p* < 0.05). Female patients who were bothered a lot by *something bad that happened recently* (adj. OR = 1.40; *p* < 0.05) and by *financial problems or worries* (adj. OR = 1.34; *p* < 0.05) were more likely to be physical inactive.

## Discussion

The overall stress score in our sample (M = 8.08) was much higher compared to primary care patients (PHQ-stress: M = 2.3 for older and M = 4.7 for younger patients; [40]. Women were significantly more bothered by psychosocial stress than men. This sex difference has also been found in previous studies [1, 28, 40]. The most frequent stressor by which both male and female patients were bothered a lot by was worries about health in almost 60% of our sample. The second most severe stressor was worries about weight or look in women and financial problems in men. However, stress at work or at school was common in both. Those were also frequent stressors found in former studies [1, 40], although differences in the order existed.

The global PHQ-stress score was significantly associated with health behaviors. After adjustment for further variables the stress score was associated with obesity, uncontrolled eating and physical inactivity in women and concerning men with smoking, uncontrolled eating and physical inactivity.

The focus on specific stressors made clear, that the associations between the stress score and health behaviors were based on certain stressors. Some specific stressors were not at all associated with health behavior. This is similar to a study by Slopen et al. (2013). The authors focused on smoking and concluded that the cumulative stress score did not predict smoking behavior better in the general population, as some stressors were not associated with it at all (such as neighborhood stress or work-family conflict) [12].

Hereafter, relevant stressors and health behaviors from our findings will be emphasized.

Financial problems or worries as well as social support (“having no one to turn to with problems”) have been identified as important stress factors being associated with health behaviors in a clinical sample.

In terms of financial problems, the adjusted regression model showed higher chances of uncontrolled eating and obesity as well as smoking and physical inactivity in women having great financial worries. Male patients had higher chances of smoking and uncontrolled eating. Financial problems showed incremental explanation of variance in health behaviors beyond known factors such as sociodemographic variables (age, education) as well as depression, which the regression model has been adjusted for. A negative effect of financial problems on smoking behavior [12] and on weight gain respectively obesity [14] has been reported in previous studies concerning the general population. Indeed, findings were not fully consistent as high financial stress has also led to higher odds of quitting smoking [12].

Another important stressor being associated with adverse health behavior was lack of social support. Being bothered a lot by having no one to turn to with problems was associated with obesity, uncontrolled eating and physical inactivity in women. In men having no one to turn to with problems was associated with uncontrolled eating and physical inactivity. A laboratory study with female students also indicated the relevance of social stress on health behavior [41]. The students showed a higher caloric intake when being confronted with an induced social stressor compared to an academic stressor. Another study found a positive association of social support with better dietary quality in persons from the general population [42]. This goes inversely in line with our findings according to eating behavior.

Interestingly, being bothered a lot by worries about health was associated with lower chances of being a smoker. Adjusted for covariates, this was only significant in men. It corresponds with a finding that many smokers do not consider themselves to be a high risk group in terms of cancer or cardiovascular disease [43]. Also, health-related worries were not associated with obesity or physical inactivity although those lifestyle behaviors have higher health risks as well. We also found stress factors that were not associated with health behaviors at all. Those were little or no sexual desire concerning both sexes. Furthermore, regarding men difficulties with partner and something bad that happened recently had no impact on health behavior. In sum, we found health behaviors to be influenced selectively by certain psychosocial stressors. Concerning health behaviors, mostly associated with psychosocial stress were uncontrolled eating in both sexes and obesity in women. Especially uncontrolled eating was mostly associated with the considered psychosocial stress factors and showed the highest effect sizes. This goes in line with previous research. The majority of previous studies reported that stress predicted uncontrolled eating [30], binge eating [33, 34] and worse dietary habits such as the intake of high caloric food [32, 44, 45]. Some studies also reported findings to predominantly eat less in stress (so called “stress-undereaters”) or found no effect on eating behavior [41, 46, 47]. Also, in previous studies [14, 27] obesity has been linked to stress rather in women than in men. Contrary to studies from the general population [20–22] we did not see that women were more vulnerable to smoking when having stress.

We only found marginal sex differences. Financial stress was associated with health behaviors in both, men and women. However, having no one to turn to was clearer associated with health behaviors in women, while stress of care taking was clearer in men. This is interesting, as women were much more often bothered by stress of care taking. Nonetheless, this psychosocial stressor seemed to affect health behavior of men more negatively. In sum and contrary to our hypothesis, sex differences were not that distinctive concerning the associations between psychosocial stress and health behaviors. One potential reason might be due to differences in the measurement as different studies measured psychosocial stress differently. It is also possible, that this illustrates differences between persons from the general population and the considered sample of patients. Further research should clarify this. Sex differences were only clear on descriptive level, where women were consistently more burdened by psychosocial stress, which goes in line with previous research [11, 28, 40, 48].

Influences on health behavior are multifactorial and we could indicate that psychosocial stress has small, but significant associations with health behaviors in a clinical sample.

### Limitations & strengths

One of the limitations of our study is the cross-sectional design which is not able to allow statements towards causality and which does not provide information on how long patients have been bothered by certain stressors. Although associations between psychosocial stress and health behaviors have been highly significant, we have mainly found low effect sizes. This has been shown for instance by the explained variance and goes in line with previously reported studies. Still we found an incremental effect of stress on health behavior beyond known factors such as demographic and socio-economic variables.

Although we considered psychosocial stress to affect health behavior, influences can be reverse as well. Moreover, limitations concerning the measurement of health behaviors should be pointed out. Due to the limitations of clinical records we were only able to conduct basic measures of health behaviors. Moreover, all measurements are self-rated, which might be a source of error. A further aspect refers to physical inactivity, which has been measured only by one item. For this reason it can only be considered as a rough screening. More detailed instruments (e.g. International Physical Activity Questionnaire, IPAQ; [49]) would have been more sophisticated. On the other side, longer scales do not seem to have an added value compared to one item scales [50, 51]. Stress-related eating for instance can include different aspects as the amount and type of food [30, 52]. We cannot answer if patients eat more or less when being bothered by psychosocial stress. We can only conclude that patients seem to eat uncontrolled when being bothered by certain stressors. The measurement of psychosocial stress is complex as different concepts exist and many assessments are available [4, 5, 53]. We measured psychosocial stress via the PHQ-stress module which includes ten major stressors. In contrast to more detailed scales [5, 54], the PHQ-stress module can only be considered as a screening instrument. It is useful to quickly identify important psychosocial stress factors in patients. The items are heterogeneous, nonetheless the scale’s reliability is still acceptable (α = 0.71).

Still, we wanted to screen for specific stressors contrary to e.g. the Perceived Stress Scale (PSS-10; [55]) which does not name particular stressors. We showed the usability of PHQ-stress being part of the most important comprehensive clinical measure (PHQ) with the external criterion of health behavior. While the Patient Health Questionnaire is a well validated instrument [56–59], the module PHQ-stress has been neglected. To our knowledge the PHQ-stress module has not been validated, however it has been used in different studies [40, 60–63]. Efforts to validate PHQ-stress would be helpful.

Health behavior is influenced by different factors, amongst others by somatic and mental illness. We did not control for comorbid somatic diseases as it is almost impossible to conclude on functional aspects just from the ICD-10 diagnosis, which can be seen as a limitation. However, health behavior is strongly affected by mental illness as depression [64–66]. In order to reduce this bias, we adjusted our analyses where necessary for depressive disorders, which were diagnosed by a clinician for all patients. We consider this as a strength of our study as we were able to adjust for the severity of depression. Where elsewhere necessary, besides demographic variables we adjusted for strong overweight of parents. By this, we roughly took genetic and environmental factors into account. A further strength is our large clinical sample and the consideration of different health behaviors. Many studies analyzed samples from the general population and we were able to close a gap for a clinical sample which is generally at high risk for maladaptive health behaviors.

## Conclusions

Longitudinal studies are necessary to further research the impact of psychosocial stress on health behaviors in mental health patients. Presumably, greater associations between those variables would be found in a longitudinal design. The duration and the severity of different stress factors as well as coping strategies should be taken into account. In addition, it would be important to consider sex differences which we found on descriptive level significantly, but not in terms of associations. These findings might be different in longitudinal studies.

As a clinical implication, specific trainings can be tested for certain clusters of psychosocial stress. For example, to reduce psychosocial stress resulting from relationships (e. g. having no one to turn to) an additional treatment to improve social skills for those patients can be beneficial. Especially, patients with financial strain and lack of social support might need specific assistance to improve their health behavior.

It would be helpful to assist patients in developing functional coping strategies which enable them to reduce unhealthy behaviors for emotional regulation. In the recent years, different ways to include treatment of unhealthy behaviors into psychotherapy were presented [67–69]. Moreover, a more holistic model of the patient’s disorder is necessary, as psychosocial stress, psychopathology and health behavior presumably influence each other mutually. This might be the case in patients seeking psychotherapeutic treatment in particular as these are more often affected by psychosocial stress than for instance primary care patients. The improvement of health behaviors should be part of the treatment in general as these patients are more likely to show unhealthy behaviors [70–72], which contribute to a worse somatic health status [73–76]. Furthermore, the improvement of health behaviors can have positive effects on mental disorders. For instance, a recent review has shown antidepressant effects of physical activity [77].

## Abbreviations

BMI: Body mass index
OR: Odds ratio
PHQ: Patient Health Questionnaire
SD: Standard deviation
ß: standardized beta
TSST: Trier Social Stress Test
